# The role of the electrocardiogram in the recognition of cardiac transplant rejection: A systematic review and meta‐analysis

**DOI:** 10.1002/clc.23783

**Published:** 2022-01-23

**Authors:** Hashim T. Hashim, Mustafa A. Ramadhan, Shoaib Ahmad, Jaffer Shah, Joseph Varney, Karam R. Motawea, Omneya A. Kandil

**Affiliations:** ^1^ College of Medicine University of Baghdad Baghdad Iraq; ^2^ Punjab Medical College Faisalabad Pakistan; ^3^ Drexel University College of Medicine Philadelphia Pennsylvania USA; ^4^ School of Medicine American University of the Caribbean St Maarten USA; ^5^ Faculty of Medicine Alexandria University Alexandria Egypt

**Keywords:** cardio transplant rejection, ECG, heart transplant rejection, rejection diagnosis

## Abstract

**Background:**

In cardiac transplant recipients, the electrocardiogram (ECG) is a noninvasive measure of early allograft rejection. The ECG can predict an acute cellular rejection, thus shortening the time to recognition of rejection. Earlier diagnosis has the potential to reduce the number and severity of rejection episodes.

**Methodology:**

A systematic literature review was conducted to identify and select the original research reports on using electrocardiography in diagnosing cardiac transplant rejection in accordance with the PRISMA guidelines. Studies included reported sensitivity and specificity of ECG readings in heart transplant recipients during the first post‐transplant year. Data were analyzed with Review manager version 5.4. *p*‐value was used in testing the significant difference.

**Results:**

After the removal of duplicates, 98 articles were eligible for screening. After the full‐text screening, a total of 17 papers were included in the review based on the above criteria. A meta‐analysis of five studies was done.

**Conclusion:**

In heart transplant recipients, a noninvasive measure of early allograft rejection has the potential to reduce the number and severity of rejection episodes by reducing the time and cost of surveillance of rejection and shortening the time to recognition of rejection.

**Abbreviations**
ECG: Electrocardiography
EMB: Endomyocardial biopsy
HT: Heart transplant
QRS, QTc, ST: ECG waves
RBBB: Right bundle branch block

## 1 INTRODUCTION

A well‐established treatment for end‐stage heart failure patients is heart transplantation. The median survival of adult patients who received a heart transplant after the year 2000 is over 12 years^1^—a significant cause of early mortality in acute allograft rejection. The prevalence of allograft rejection has been reported to exceed 13% in the first year following adult heart transplantation. Thus, if the patient survives the first‐year post‐transplant, they are expected to survive at least 15 years.^1^ According to the 2011 annual United States data released by the International Society for Heart Lung Transplantation Registry, 26% of heart transplant patients experience at least one rejection episode within the first‐year post‐transplant. The most frequent cause of morbidity and rehospitalization in this patient population remains acute rejection.^2^, ^3^

The electrocardiogram (ECG) is a simple, cost‐effective, and noninvasive tool used to evaluate the rhythm and electrical activity of the heart. Sensors attached to the skin are used to display the electrical signals generated by your heart on an easy to interpret grid paper.^4^ Utilizing ECG readings in heart transplant recipients can predict an acute cellular rejection, thus shortening the time to recognize rejection. A recent study examined serial ECGs in 98 patients within the first‐year post‐heart transplantation. The most common abnormalities were associated with intraventricular conduction delays, with the right bundle branch block (RBBB) being the most prevalent.^5^, ^6^

In cardiac transplant recipients, a noninvasive measure of early allograft rejection can reduce the number and severity of rejection episodes. ECG can reduce the time to detection and the cost of surveillance of rejection.^6^ In this study, we summarize the diagnostic accuracy and criteria of the ECG in the detection of cardiac transplant rejection patients.

## 2 METHODOLOGY

### 2.1 Selection of studies

A systematic literature review was conducted to identify all studies about the detection of graft rejection in heart transplant surgeries per the PRISMA guidelines.^7^ The online database: Google Scholar, PubMed, and Cochrane were searched from January 1985 to September 2020. Keywords used in the search included (Heart transplant rejection OR Heart transplantation rejection OR Detection of heart transplant rejection OR Cardiac transplant detection OR Cardiac transplantation detection OR Noninvasive ways of detection of cardiac transplant rejection). The screening was completed by Hashim T. Hashim, and Jaffer Sha, with disagreements being resolved by Joseph Varney. There was no restriction on participant's age, gender, or ethnicity, and no restrictions to language written. The references of selected papers were manually checked for additional relating studies. An analysis of the funnel plot was carried out to determine the possibility of bias in the publication in the Review Manager program version 5.4. Inclusion criteria for meta‐analysis were studies that correlated the Endomyocardial biopsy grading to the ECG features.

### 2.2 Data extraction

Details of the study design, ECG characteristics, endomyocardial biopsy grading, and outcome data, including the QT interval, QTc, QT dispersion, and QTc dispersion, were extracted. The risk of bias was assessed using the Newcastle‐Ottawa Quality Assessment tool.

### 2.3 Study inclusion

**Figure 1:**
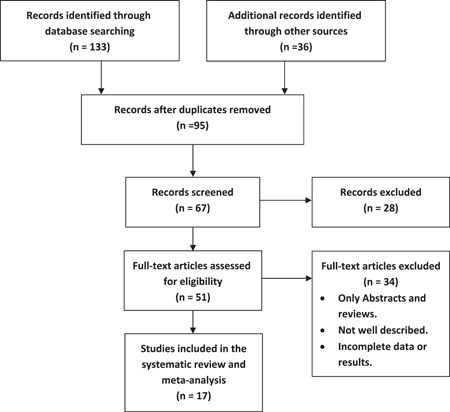
Flow chart of the study selection process

After a comprehensive search of the literature, 170 publications resulted and then became 96 after removal of duplicates. Of these, 51 were eligible for full‐text screening. After the full‐text screening, 18 studies were included in the systematic review and meta‐analysis, as shown in (Figure 1). Six studies were included in the meta‐analysis. The included QT (ms), QTc, QT dispersion, and QTc dispersion outcomes in the meta‐analysis were reported in 2, 3, and 5 studies. The summary of the included studies and risk of bias assessment are shown in Tables 1, 2, 3, respectively.

**Table 1:** The studies' general information

The code	The study	References	Year	Country
E 1	Haberl et al.	^8^	1987	Germany
E 2	Lacroix et al.	^9^	1992	Canada
E 3	Picano Et al.	^10^	1990	Italy
E 4	Regueiro‐Abel et al.	^11^	2002	Spain
E 5	Kim et al.	^12^	2019	South Korea
E 6	Imamura et al.	^13^	2012	Japan
E 7	Babuty et al.	^14^	1994	France
E 8	Doering et al.	^15^	2012	USA
E 9	Grace et al.	^16^	1991	UK
E 10	Graceffo et al.	^17^	1996	USA
E 11	Grauhan et al.	^18^	1993	Germany
E 12	Hicky et al.	^19^	2018	USA
E 13	Locke et al.	^20^	1989	UK
E 14	Vogt et al.	^21^	1990	Germany
E 15	Tenderich et al.	^22^	2006	Germany
E 16	Valentino et al.	^23^	1992	USA
E 17	Wada et al.	^24^	1999	Japan
E 18	Eckart et al.	^25^	2005	USA

**Table 2:** The studies' specific data

Code	Study type	No. of patients	Mean ages	Duration
E 1	Observational	19 Patients	40.46 years	14 days
E 2	Observational	25 Patients	44.2 years	5.2 months
E 3	Observational	14 Patients	48.6 years	24 h
E 4	Observational	31 Patients	‐	15 months
E 5	Retrospective analysis	79 Patients	33.6 years	5 years
E 6	Case report	2 Patients	26 years	I year
			48 years	
E 7	Review	‐	‐	‐
E 8	Prospective, double‐blind, multi‐center descriptive study	325 Patients	‐	6 weeks
E 9	Cross‐sectional	18 Patients	49 years	19 days
E 10	Observational	20 Patients	47 years	8 months
E 11	Experimental	40 Patients	‐	4.5 days
E 12	Observational	220 Patients	54 years	72 h
E 13	Observational	10 Patients	38.2 years	‐
E 14	Retrospective Study	13 Patients	49 years	3 years
E 15	Observational	100 Patients	‐	3 months
E 16	Prospective Study	41 Patients	‐	‐
E 17	Experimental	‐	‐	6 days
E 18	Cohort	75	55.2 years	7 days

**Table 3:** The ECG's characteristics

Code	ECG changes	No. of rejection	Outcomes or notes
E 1	The frequency content of the ST section decreased from 10 to 30 Hz, and the frequency content of the QRS varied from 60 to 150 Hz.	16 Patients	For the noninvasive diagnosis of acute cardiac rejection after cardiac transplantation, FFT of surface ECGs is encouraging. The mechanism of improvements and the future application of this approach for persistent rejection assessment continue to be assessed.
E 2	In detecting rejection, the root‐mean‐square voltage of the 70‐Hz high pass filtered QRS complex was found to be the most reliable component.	20 Patients	In the control of heart transplant rejection, the signal‐averaged ECG is useful. Compared with the time‐domain method, frequency domain analysis of the QRS complex would not improve the technique's precision.
E 3	Depression of the ST section in the precordial segments (mostly V3‐V6) and the limb leads.	‐	Dipyridamole electrocardiography in the early post‐transplantation era is practical, secure, and affordable, with the potential for noninvasive monitoring of transplantation rejection.
E 4	In the AR group, the QTc dispersion was 40 ± 17 ms.	31 Patients	Proposals for the use of QTc dispersion for diagnosing AR in HT patients are not confirmed by the findings of this review.
E 5	Longer PR interval RBBB ECG changes.	3 Patients	Near observation of new RBBB growth at 1‐year post‐HT, associated with a higher rate of new‐onset graft rejection, can be helpful in detecting high‐risk graft rejection patients.
E 6	Disappearance of R waves in I, aVL, and prolongation of wider QRS duration PR intervals and deeper S waves in V5,6 in I, II, aVF deep S wave, poor R progression in all anterior precordial leads, marked PR interval prolongation, and V4‐6 deeper S wave. Also found were ST depression and T wave inversion in I, aVL, and V2‐6.	2 Patients	While no rejection‐specific ECG changes have been reported so far, the above‐described changes that may represent actual hemodynamic anomalies may be a diagnostic tool for rejection.
E 7	Important changes in the high‐frequency components (between 50 and 110 Hz) of the QRS complex and significant reductions in the low‐frequency components (between 10 and 30 Hz).	‐	During acute rejection, improvements in the ECG properties of transplanted hearts were observed, with improvements in intraarticular and auriculoventricuir conduction and decreases in QRS voltage amplitude. These experimental findings should be considered in the development of new methods for detecting cardiac allograft rejection ECGs.
E 8	An expanded QTC interval in recipients of a heart transplant is linked to acute allograft rejection and death.	‐	In heart transplant recipients, a noninvasive measure of early allograft rejection has the ability to reduce the number and severity of rejection episodes by reducing the time and cost of monitoring of rejection and shortening the time to identification of rejection. In addition, other ECG parameters important to noninvasive allograft rejection monitoring must be identified to achieve the objectives of the current study and may provide evidence for a randomized controlled trial to assess the feasibility and cost‐effectiveness of this form of noninvasive ECG monitoring as compared with normal EMB surveillance.
E 9	A decrease in the summed QRS voltage in the anterior chest leads and a turn to the right in the QRS frontal vector was also seen in humans and nonspecific repolarization shifts were also seen and drops in the evoked T wave amplitude.	18 Patients	At the time of transplantation, QT‐driven rate‐responsive units can be implanted with periodic interrogation of these units, theoretically abrogating the need for endomyocardial biopsy.
E 10	Higher frequency QRS voltages.	20 Patients	The study shows the relative loss of high‐frequency SA‐ECG components in cardiac transplant rejection patients and suggests that SA‐ECG may be useful for noninvasive cardiac transplant rejection assessment.
E 11	ECG voltage amplitude (IMEG) seems to follow a "focal pattern" similar to the histology.	40 Patients	‐
E 12	Important changes in the length of QRS (*p* < .001), QT (*p* = .009), QTc (*p* = .003), and PR (*p* = .03) cycles, as well as increased odds of development of right bundle block branch (*p* = .002) and fascicular block (*p* = .009).	12 Patients	Electrocardiographic changes following transplant surgery have been linked with mild to serious acute allograft rejection.
E 13	Significant decreases in QRS voltage.	10 Patients	These results suggest that in the estimation of cardiac rejection, QRS voltage is of highly limited benefit in patients treated with low‐dose triple immunotherapy.
E 14	QRS reduction in the standard ECG.	13 Patients	A useful screening tool for mild to extreme acute rejection is QRS voltage reduction in a localized region measured by BSPM.
E 15	Prolongation in both QTc time and QTc dispersion of >40 ms.	100 Patients	ECGs are routinely conducted, QTc time measurements and QTc dispersion can be accurately used to detect acute rejection early after heart transplantation.
E 16	A significantly larger high frequency QRS complex component (50–110 Hz).	19 Patients	ECG for the diagnosis of acute allograft rejection is a useful noninvasive technique.
E 17	The QRS complex's peak‐to‐peak amplitudes and heart rate are substantially reduced, the power of and the LF is significantly increased.	‐	A successful noninvasive marker for early detection of cardiac allograft rejection is heart rate variability study. A responsive means of measuring the effects of immunosuppressive therapy can also be given by this procedure.
E 18	Increased QT dispersion in patients with rejection.	41	No statistical significance of QTc−d and severity of rejection. QTc−d should not be considered a sensitive marker for OHT rejection.

### 2.4 Analyses

The total number of patients included in the meta‐analysis in the no rejection or mild rejection group is 1733 patients, and the total number of patients in the moderate or severe rejection group is 264 patients.

## 3 RESULTS

We used random effects due to heterogeneity observed among studies when we used fixed effects. In QT (ms) outcome, the pooled analysis between no or mild rejection and moderate or severe rejection was (MD = 3.80, 95% CI = −18.10 to 25.70, *p*‐value = .73), we observed heterogeneity that was not solved by random effects, as shown in Figure 1. The pooled analyses between no or mild rejection and moderate or severe rejection in QTc, QT dispersion and QTc dispersions outcomes, were (MD = 18.91, 95% CI = −21.30 to 59.11, *p*‐value = .36), (MD = −68.54, 95% CI = −195.74 to 58.66, *p*‐value = .29) and (MD = −41.15, 95% CI = −93.26 to 10.96, *p*‐value = .12), respectively (Figures S2–S4).

We did subgroup analysis based on the duration of the follow‐up. The two subgroups were from 3 to 6 months and from hospital discharge to 7 days, the heterogeneity was not solved by subgroup analysis and leave one out test in the QTC subgroups and the results (MD = 5.19, 95% CI = −15.55 to 25.92, *p*‐value = .62) and (MD = 34.67, 95% CI = −30.99 to 100.33, *p*‐value = .30), respectively (Figure S5).

In the QTc dispersion outcome, the heterogeneity was not solved by subgroup analysis or leave one out test. The results were in the 3–6 months follow‐up subgroup (MD = −68.77, 95% CI = −195.54 to 58.01, *p*‐value = .29) and in the hospital discharge to 7 days follow‐up subgroup were (MD = 10.00, 95% CI = − 2.17 to 22.17, *p*‐value = .11) (Figure S6).

No publication bias was observed among included studies, as shown in Figure S7.

Table 1 describes the characteristics data for each study individually and their citations.

Table 2 shows the data of patients and their characteristics, their ages, the type of study, and the duration of the study (the age and the duration are either mean or median).

Table 3 shows the characteristics of the ECG recording and the outcomes of the studies (No. of rejections is the number of patients recognized with ECG).

**Figure 2:**
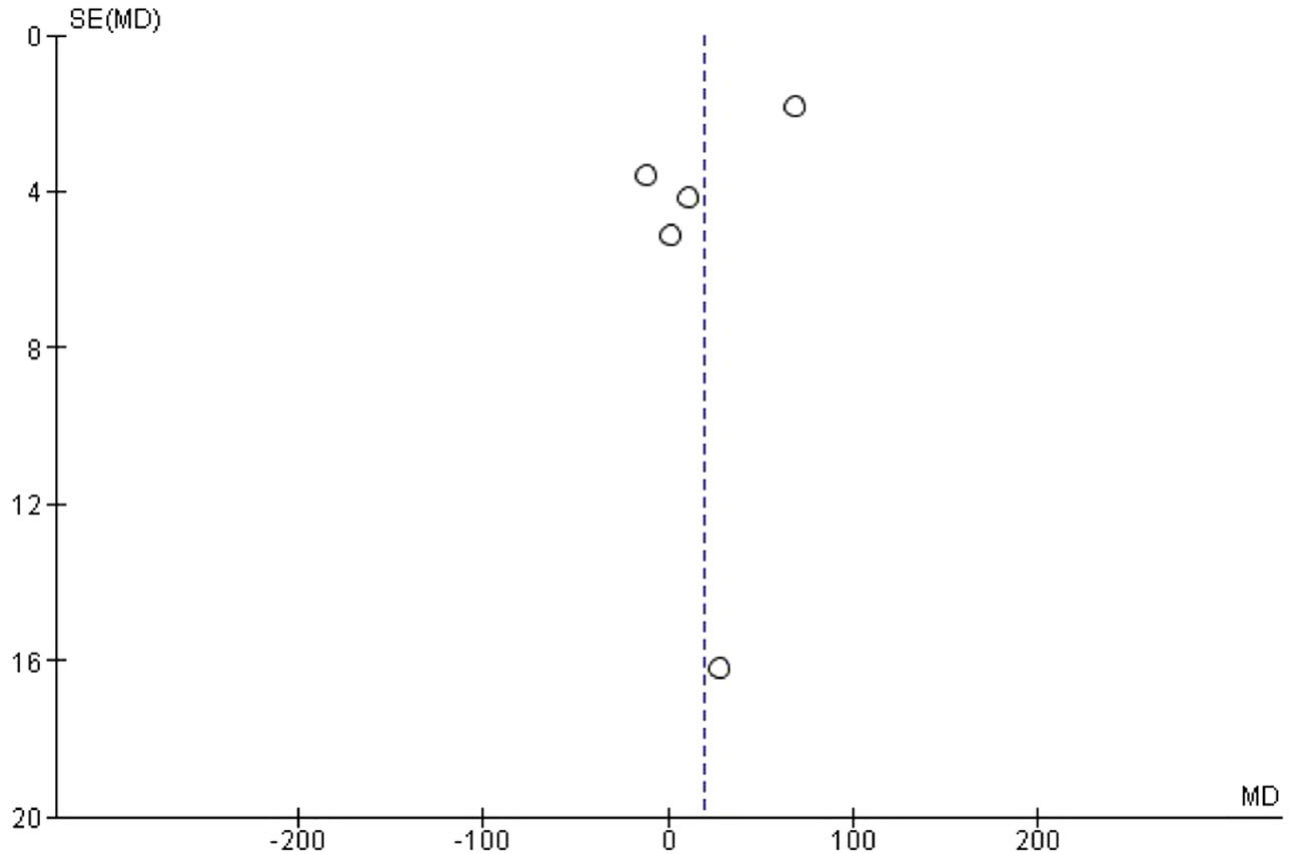
Publication bias

The rejection was diagnosed with histology findings and biopsies and then compared with the findings of the ECG to give the definitive diagnosis (Figure 2).

Figure S7 shows the risk of biases and applicability concerns among the studies distributed as high risk, low risk, and unclear risk.

## 4 DISCUSSION

We found no significant association between heart transplant rejection and QT changes of ECG. The studies included in this review report the rejection of the heart transplant after the surgery with either moderate or severe rejection. The results were assured by the biopsy to compare between the results of the ECG and the histology. A total of 957 patients were identified for heart transplant rejection, with 304 diagnosed by ECG (31.7%). The primary method used for diagnosis was the QRS interval and amplitude (see Table 3). Sensitivity and specificity varied widely between our studies (see Figure S3), potentially showing the user error in ECG placement and reading. To date, the only consistently effective approach available for the diagnosis of cardiac transplant rejection is an endomyocardial biopsy.

The utility of ECG in this population may have various utilities. Preclinical advances in cardiac transplantation have shown that ECG may indicate a beneficial corticosteroid response.^26^ After cardiac transplant, the incidence of conduction disorders is well known, and RBBB is the most frequent of these.^27^ The occurrence of RBBB within 1 month of cardiac transplant might have different clinical consequences from those with later RBBB incidence. Before the ECG can consistently be used to detect acute allograft rejection, an investigation is still required to assess computerized ECG measurement algorithms.

Given that ECGs are carried out regularly, QTc time and QTc dispersion measurements could be used to accurately detect acute rejection at the early stage after heart transplantation. If further studies confirm our early current findings, it will be possible to implant QT‐driven rate‐sensitive units with periodic interrogation of these units at the time of transplantation. This could nullify the need for endomyocardial biopsy.

## 5 LIMITATIONS & FUTURE DIRECTION

There is heterogeneity among included studies due to diversity of study designs in the studies included in the meta‐analysis. Few numbers of studies are included in the meta‐analysis due to few data published. Most of the published data are about QT changes with no interest to the other components of the ECG. ECG abnormalities are less sensitive to the mild forms of rejection that occurs with the currently used immunosuppression medications. The use of the SA‐ECG may be combined with the help of the standard ECG to not miss patients with milder rejection who do not have abnormal ECG features.^28^ Further studies are needed to determine if the frequency or time domain of the SA‐ECG are better predictors of rejection.

## 6 CONCLUSION

In heart transplant recipients, the ECG is a noninvasive measure of early allograft rejection. It holds the potential to reduce the number and severity of rejection episodes of rejection. Time‐efficient and low cost make the ECG a good choice for screening, yet specificity and sensitivity varied widely throughout the studies chosen. Reasonings behind this could be as simple as user error. To avoid this shortfall, an algorism for artificial intelligence reading cardiac transplant rejection patients should be created. With the exceedingly high cost of a heart transplant, we feel further investigation is warranted.

We found no significant association between heart transplant rejection and QT changes of ECG. Although some studies reported significant association, other studies did not. There is heterogeneity among studies included in the meta‐analysis, that does not provide conclusive results. More clinical trials are needed to give final conclusion about using ECG as a measure in detecting heart transplant rejections.

## CONFLICT OF INTERESTS

The authors declare that there are no conflict of interests.

## CONSENT FOR PUBLICATION

All the authors provided their consents for publication.

## AUTHOR CONTRIBUTIONS

Hashim T. Hashim and Mustafa A. Ramadhan created the idea, wrote the first draft and supervised the work; Joseph Varney, Jaffer Shah, and Shoaib Ahmad did the data collection and studies selection; Karam Ramadan Motawea, Omneya A. Kandil, and Joseph Varney wrote the final draft and did the analysis.

## Supporting information

Supplementary information.

## Data Availability

Data can be requested from the corresponding author upon a reasonable request. This manuscript is being submitted on behalf of all authors listed. The authors declare that this manuscript is solely being submitted to your journal and is not currently under review by any other journal.
