# Supplementary material for: The role of the electrocardiogram in the recognition of cardiac transplant rejection: A systematic review and meta‐analysis
Source: Clin Cardiol. 2022 Jan 23;45(3):258–64. doi: 10.1002/clc.23783 (PMC8922543; doi:10.1002/clc.23783)
Supplement: Supplementary file 1 — Supplementary information. [file CLC-45-258-s001.docx]

**Supplementary Figure 1. Forest plot of the QT (ms) outcome**


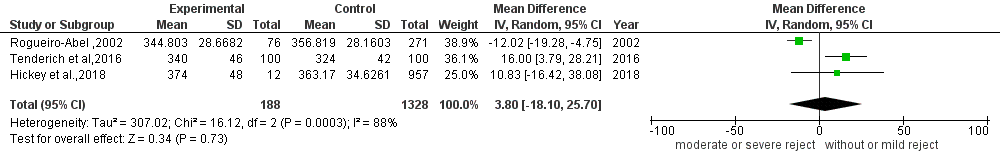


**Figure 2. Forest plot of the QTc outcome**


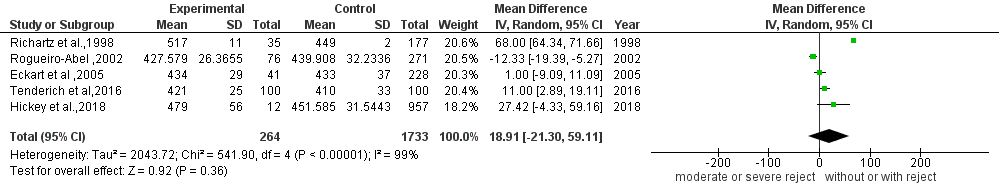


**Supplementary Figure 3. Forest plot of the QT dispersion outcome**


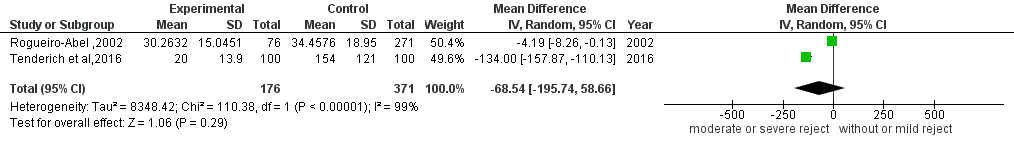


**Supplementary Figure 4. Forest plot of the QTc dispersion outcome**


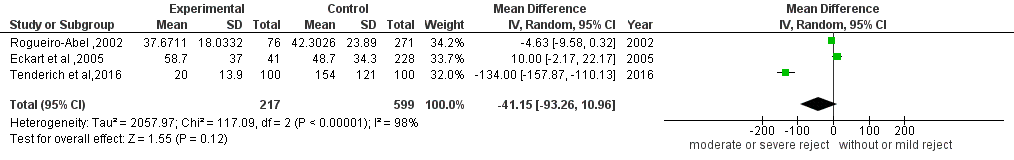


**Supplementary Figure 5. Forest plot of the QTc subgroup analysis**


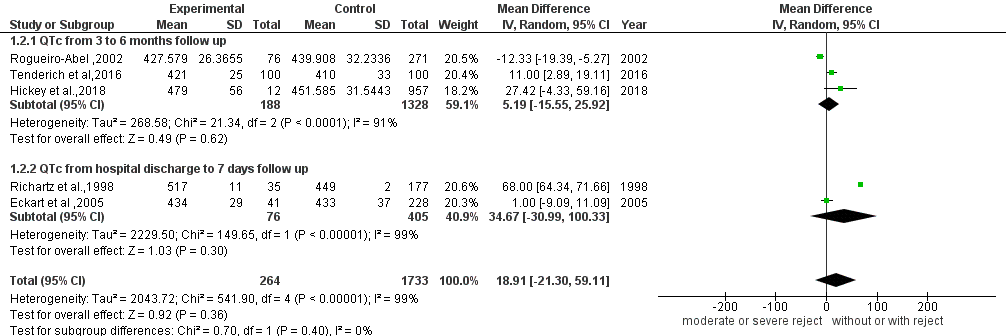


**Supplementary Figure 6. Forest plot of the QTc dispersion subgroup analysis**


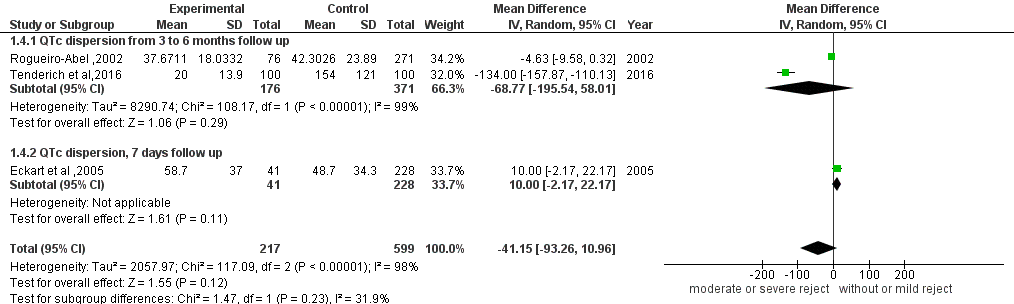


**Supplementary Figure 7. The biases risk assessment**

**
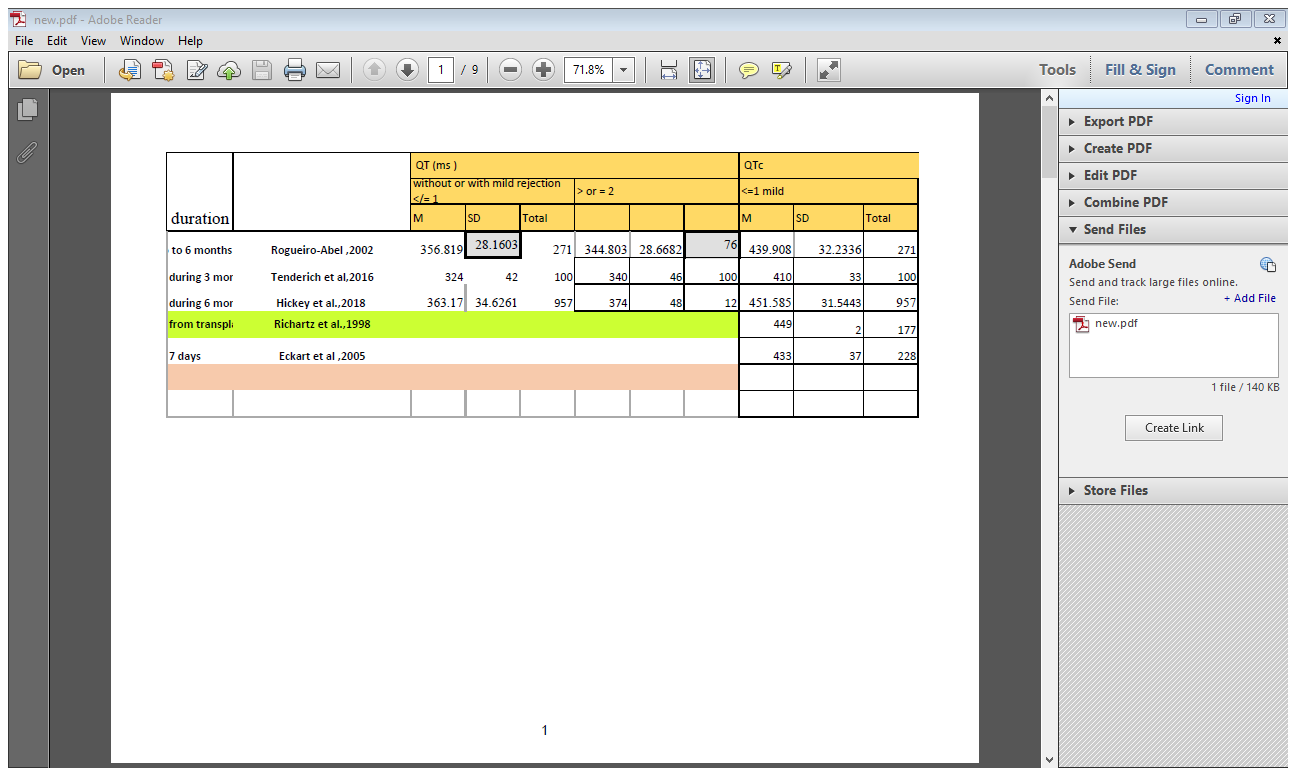
**
